# Paternal age impairs in vitro embryo and in vivo fetal development in murine

**DOI:** 10.1038/s41598-022-16469-9

**Published:** 2022-07-29

**Authors:** Larissa Araújo Stábile, Camilla Mota Mendes, Marcelo Demarchi Goissis, Raphaela Gabrielle Brito Sousa, Marcílio Nichi, José Antônio Visintin, Thais Rose dos Santos Hamilton, Mayra Elena Ortiz D’ Ávila Assumpção

**Affiliations:** Department of Animal Reproduction, School of Veterinary Medicine and Animal Science, University of Sao Paulo, Sao Paulo, 05508 270 Brazil

**Keywords:** Senescence, Embryology

## Abstract

The association between advanced paternal age and impaired reproductive outcomes is still controversial. Several studies relate decrease in semen quality, impaired embryo/fetal development and offspring health to increased paternal age. However, some retrospective studies observed no alterations on both seminal status and reproductive outcomes in older men. Such inconsistency may be due to the influence of intrinsic and external factors, such as genetics, race, diet, social class, lifestyle and obvious ethical issues that may bias the assessment of reproductive status in humans. The use of the murine model enables prospective study and owes the establishment of homogeneous and controlled groups. This study aimed to evaluate the effect of paternal age on in vitro embryo development at 4.5 day post conception and on in vivo fetal development at 16 days of gestation. Murine females (2–4 months of age) were mated with young (4–6 months of age) or senile (18–24 months of age) males. We observed decreased in vitro cleavage, blastocyst, and embryo development rates; lighter and shorter fetuses in the senile compared to the young group. This study indicated that advanced paternal age negatively impacts subsequent embryo and fetal development.

## Introduction

Parenthood has been delayed, a possible explanation is the inversion of life goals, men and women are prioritizing specialized education and career stability, furthermore has the financial considerations, health implications, low reception of labor market for mothers and the notoriety of assisted reproductive technologies which is seen has a security concerning to late conception^1^. The decline in female reproductive potential over time is widely studied. In fact, women’s age is related to decreased fertility and offspring abnormalities^2,3^ such as trisomy^4^, birth defects^5^, and reduced fetal growth^6^. Recently, there is an increasing concern regarding the effect of age on male reproductive outcomes^7–9^.

It is important to highlight there is no consensus among researchers about advanced paternal age and its consequences. Some meta-analysis studies suggested that the increased paternal age does not influence fertilization rates, in vitro embryo development, and embryo implantation and pregnancy^10^. Also, there are studies suggesting that increased paternal age does not influence sperm parameters such as morphology^11^, motility, or concentration^12^. However, the majority of the studies showed that older men have sperm alterations, such as an increase in abnormal cells^13^, a decrease in sperm motility^14^; conditions correlated with a delayed gestation and decreased fertilization capacity^15^. In addition, pregnancy rates from men older than 40 years of age are 10% lower when compared to those from men younger than 24 years of age^16^. Paternal aging was also suggested to be related to a progressive decrease in sperm quality, subfertility, and infertility that were correlated with problems in offspring^17^, such as neurocognitive disorders schizophrenia^18^, autism^19^ and bipolar disorders^20^, as well as chromosomal errors^21^, decreased pregnancy rate^22^, fetal deaths^8^, placental growth, and development^23,24^.

These published studies were mostly retrospective and performed in assisted human reproduction clinics and used different cut-off points to denote advanced paternal age, which may have interfered in systematic assessments of reproductive risk associated with paternal aging. Since the twentieth century, scientific research has used the mouse model as pointed in Table 1. Thus, endorsing and justifying the use of the murine model to infer changes in humans and offers the opportunity to study several physiological mechanisms, including reproductive physiology, in contrast to other species. Moreover, Dutta and Sengupta correlated the age of the human species with the murine species, which enabled the establishment of the murine experimental models^34^.

**Table 1:** Summary of advantages for use murine as a model in scientific research.

Observations	References
Anatomical and physiological similarities with humans	Bryda^25^, Uhl^26^, Vandamme^27^
Easily handling on laboratory animal facility	Bryda^25^, Uhl^26^, Vandamme^27^
Short generational interval and larger litter size	Bryda^25^, Uhl^26^, Vandamme^27^
Use of inbred strains promote homogenous control group on a controlled environment	Casellas^28^ and Barré-Sinoussi et al.^29^
Full genome sequenced	Waterston et al.^30^
Model for male infertility	O'Bryan^31^, Jamsai et al.^32^ and Godmann et al.^33^

Murine males at 27 months of age presented decreased reproductive efficiency observed by an increase of mating interval, compared to males at middling 3, 16, and 23 months of age; moreover, offspring from females mated with males at 27 months of age presented less longevity^35^, this represents approximately in humans 10, 54, 77 and 90 years of age^34^. Another study observed reduced straightening reflex, spontaneous motor activity, and passive avoidance learning on offspring from males at 27 months of age^36^.

Therefore, studying senile murine males, between 18 and 24 months of age, will be important, as studies with advanced paternal age does not prospectively explore the relationship of possible seminal alterations with changes on embryo and fetal development. We tested the hypothesis that senile male mice mated with females at a reproductive age of 2–4 months, negatively affects in vitro embryo and in vivo fetal development. This study aimed to evaluate in vitro embryo development and fetal development at day 16 of gestation from female mice mated with young and senile males.

## Results

### Reproductive performance of murine males

**Figure 1:**
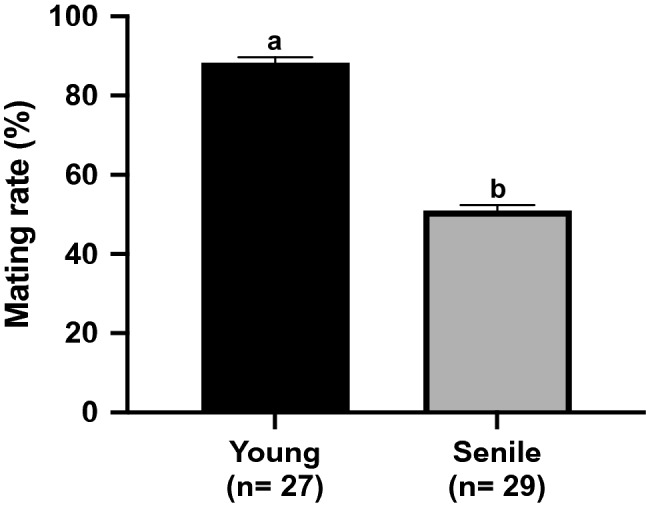
Mating rate. Reproductive performance of young (n = 27) and senile (n = 29) groups. Different superscript letters in each bar represent p < 0.05, as indicated by T-test.

We observed a lower mating rate (number of females with vaginal plug/total number of females that copulated × 100) in senile compared to the young group (50.85 ± 7.77% and 88.33 ± 7.26%; p = 0.0126; Fig. 1).

### In vitro embryo development

**Figure 2:**
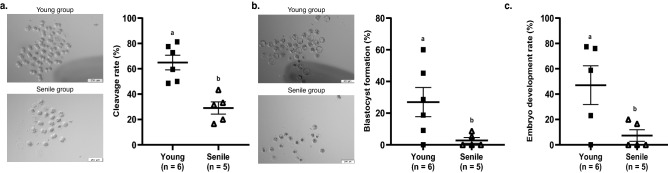
In vitro embryo development. Image and chart of cleaved embryos from the young and senile group (number of cleaved embryos/number of total structures × 100; **a**). Image and chart of blastocyst from the young and senile group (number of blastocysts/number of total structures × 100; **b**), and embryo development rates (number of blastocysts/number of cleaved embryos × 100; **c**) from young and senile groups. In the young group: 185 total structures (presumed zygotes) from 6 pregnant dams (30.8/pregnant dam), 122 cleaved embryos from 6 pregnant dams (20.3/pregnant dam), and 61 blastocysts from 6 pregnant dams (10.1/pregnant dam). In senile group: 107 total structures from 5 pregnant dams (21.4/pregnant dam), 27 cleaved embryos from 5 pregnant dams (5.4/pregnant dam) and 3 blastocysts from 5 pregnant dams (0.6/pregnant dam). The evaluations were performed in stereomicroscope (Olympus SZ61, Olympus^®^, Tokyo, Japan) at ×60 magnification. Different superscript letters in each bar represent p < 0.05, as indicated by statistical T-test.

On day 1.5 in vitro culture (IVC), senile males sired embryos that had lower cleavage rates (number of cleaved embryos/number of total structures × 100: 29.00 ± 4.83% and 64.92 ± 5.78%; p = 0.0012; Fig. 2a) compared to young males. On day 4.5 the percentage of blastocysts was reduced (number of blastocysts/number of total structures × 100: 2.79 ± 1.79% and 26.94 ± 9.20%; p = 0.0465; Fig. 2b) and embryo development rate was lower (number of blastocysts/number of cleaved embryos × 100: 7.33 ± 4.52% and 58.82 ± 12.64%; p = 0.0207; Fig. 2c) in senile compared to young, respectively.

### Fetal development

**Figure 3:**
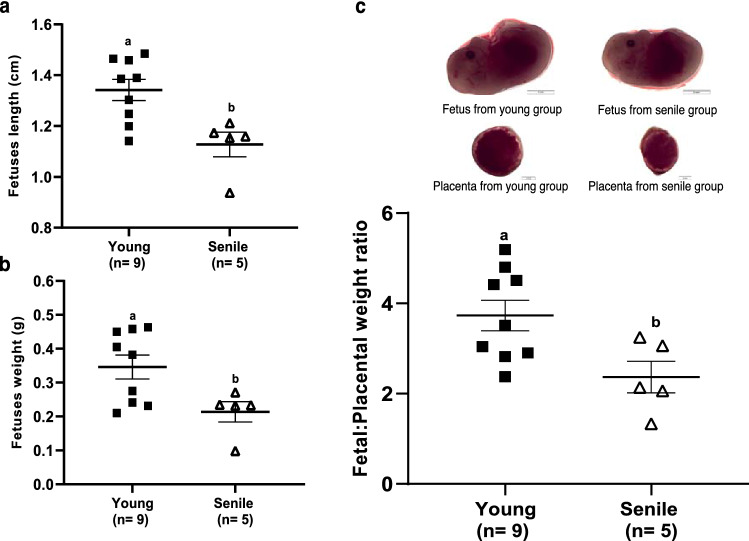
Fetal development at 16 days of gestation. Fetuses length (**a**) and weight (**b**), and fetal: placental weight ratio (**c**) of young and senile groups. We considered the average of the litters per male, 14 litters as experimental unit (5 litters in the senile group and 9 litters in the young group). The photos were performed with a megapixel digital color camera (Olympus LC30, Olympus^®^, Munster, German) attached to a stereomicroscope (Olympus SZ61, Olympus^®^, Tokyo, Japan) at ×8 magnification. Different superscript letters in each bar represent p < 0.05, as indicated by statistical T-test.

Fetuses from females mated with senile males were smaller (1.127 ± 0.048 cm and 1.34 ± 0.041 cm; p = 0.0074; Fig. 3a) and lighter (0.213 ± 0.029 g and 0.346 ± 0.035 g; p = 0.027; Fig. 3b) than the fetus from the young group. Also, fetus placental weight ratio decreased on senile group (2.368 ± 0.351 and 3.730 ± 0.338; p = 0.023; Fig. 3c) compared to the young group, respectively.

No differences were observed between groups for placental weight (0.094 ± 0.012 g and 0.092 ± 0.003 g; p = 0.90), length (0.769 ± 0.050 cm and 0.771 ± 0.021 cm; p = 0.96) and area (0.458 ± 0.072 cm^2^ and 0.455 ± 0.022 cm^2^; p = 0.96). Similarly, no difference in the in the number of total structures (12.4 ± 1.66 and 15.11 ± 2.01; p = 0.38), number of viable fetuses (7.5 ± 1.97 and 8.11 ± 0.69; p = 0.72), or in number of resorptions sites (4.90 ± 1.34 and 7.00 ± 2.07; p = 0.49) were observed between senile and young groups, respectively.

### Sperm evaluation

There was no difference between the young and senile groups for the analyses performed by flow cytometry (mitochondrial membrane potential, plasma and acrosome membrane integrity, and oxidative stress; see Table 2). Considering CASA analysis, we observed lower average pathway velocity (VAP) straight-line velocity (VSL); curvilinear velocity (VCL); total sperm motility (TM), percentage of sperm with progressive motility (PROG), percentage of rapid sperm (RAPID) in the senile group compared to the young group, as shown on Table 2. Moreover, a higher percentage of static sperm (STATIC) was observed in the senile group compared to the young group (Table 2). No differences were observed in the other variables evaluated by CASA between the groups (Table 2).

**Table 2:** Sperm Evaluation performed by flow cytometry and computer-assisted sperm analysis (CASA). *VAP* average pathway velocity, *VSL* straight-line velocity, *VCL* curvilinear velocity, *ALH* amplitude of lateral head displacement, *BCF* beat cross frequency, *STR* straightness, *LIN* linearity, *TM* total motility, *PROG* progressive motility, *RAPID* rapid velocity, *MEDIUM* medium velocity, *SLOW* slow velocity, *STATICS* non-moving sperm. Different superscript letters in each bar represent p < 0.05, as indicated by statistical T-test.

Sperm evaluation	Variable	Young (n = 4)	Senile (n = 5)	*p*
Flow cytometry	High mitochondrial membrane potential	30.80 ± 3.96	21.02 ± 2.50	0.06
Flow cytometry	Medium mitochondrial membrane potential	48.95 ± 1.28	41.26 ± 5.98	0.07
Flow cytometry	Low mitochondrial membrane potential	20.25 ± 4.04	37.70 ± 6.56	0.27
Flow cytometry	Intact membrane and damaged acrosome	8.46 ± 6.76	1.28 ± 0.06	0.36
Flow cytometry	Damaged membrane and damaged acrosome	21.81 ± 8.76	4.91 ± 0.30	0.14
Flow cytometry	Intact membrane and intact acrosome	27.12 ± 3.49	40.82 ± 5.70	0.09
Flow cytometry	Damaged membrane and intact acrosome	42.60 ± 12.03	52.98 ± 5.39	0.42
Flow cytometry	Intact membrane without oxidative stress	16.50 ± 1.10	24.60 ± 3.96	0.27
Flow cytometry	Intact membrane with oxidative stress	19.60 ± 4.00	13.34 ± 3.25	0.33
Flow cytometry	Damaged membrane without oxidative stress	56.90 ± 10.00	58.56 ± 2.96	0.82
Flow cytometry	Damaged membrane with oxidative stress	7.02 ± 4.88	3.47 ± 1.08	0.31
CASA	VAP (µm/s)	73.80 ± 4.44^a^	55.62 ± 3.10^b^	0.01
CASA	VSL (µm/s)	43.60 ± 3.79^a^	29.42 ± 2.15^b^	0.01
CASA	VCL (µm/s)	164.02 ± 6.30^a^	138.20 ± 4.08^b^	0.009
CASA	ALH (µm)	9.5 ± 0.10	8.86 ± 0.49	0.26
CASA	BCF (Hz)	39.00 ± 0.87	41.72 ± 1.18	0.10
CASA	STR (%)	52.75 ± 1.88	50.40 ± 1.02	0.28
CASA	LIN (%)	27.75 ± 1.93	25.00 ± 1.14	0.23
CASA	TM (%)	58.50 ± 2.93^a^	43.80 ± 4.21^b^	0.02
CASA	PROG (%)	20.00 ± 3.00^a^	8.80 ± 1.24^b^	0.007
CASA	RAPID (%)	35.75 ± 3.56^a^	18.60 ± 2.37^b^	0.004
CASA	MEDIUM (%)	22.75 ± 3.22	25.00 ± 2.30	0.57
CASA	SLOW (%)	0.75 ± 0.47	1.80 ± 0.37	0.12
CASA	STATIC (%)	40.75 ± 2.17^a^	54.80 ± 4.50^b^	0.03

### Correlation

We observed positive correlations between high mitochondrial membrane potential and cleavage (rho 0.88), blastocyst (rho 0.74), and embryo development rates (rho 0.77) in this study. For the variables assessed by CASA, we observed positive correlations between the percentage of motile sperm (TM—%) and cleavage (rho 0.69), blastocyst (0.85), and embryo development (0.78) rates. Similarly, the percentage of progressive sperm correlated positively with cleavage (rho 0.86), blastocyst (0.79), and embryo development (0.78) rates. The percentage of rapid sperm was also positively correlated with cleavage (rho 0.86), blastocyst (0.81), and embryo development (0.79) rates (Supplementary Table S1). When the correlation was performed only in the senile group, we observed a high positive correlation between high mitochondrial membrane potential with blastocyst (rho 0.89) and embryo development rates [(0.89—Supplementary Table S2)].

## Discussion

In our study, we tested the hypothesis that the in vitro embryo and in vivo fetal development was negatively affected when senile mice are mated with reproductive-aged females. In fact, our results demonstrated that females mated with senile males presented lower cleavage, blastocyst, and embryo development rates compared to the ones mated with young males.

In the present study, we observed that senile male mice had a lower cleavage and blastocyst rate than young male mice. This data corroborates with the study performed by Katz-Jaffe et al.^37^, these authors evaluated in vitro embryo development from superovulated mice females and found that males aged 12–15 months showed a decrease in the formation of the blastocyst (71% vs. 81%) and quality of morphology (Gardner and Schoolcraft grading system) of expanded blastocyst (63% vs. 71%) when compared to male mice less than 12 month of age, showing that advanced paternal age has negative effects on embryonic development. However, authors did not report differences in cleavage rate and embryo quality in this stage.

In humans, Klonoff-Cohen et al. conducted a prospective study with 221 couples during in vitro fertilization protocol and observed that each additional year of male age was associated with an additional 11% chance of not getting pregnant and 12% of unsuccessful births^38^. Nevertheless, Wu et al. did not observe any differences in fertilization and cleavage rates, embryo quality, and miscarriage rate when analyzing 9991 cycles of in vitro fertilization regardless of maternal (30–38 years) or paternal (30–42 years) age considered in this study^39^.

In the present experiment, we observed low embryo production rates probably due to the culture medium used in the in vitro manipulations. However, we believe that this factor did not influence the statistical results regarding the lower embryo production rates found in the senile when compared to the young males since this condition influenced both groups equally.

In this study, we observed that fetuses from females mated with senile males were lighter and smaller. It was described that men over 50 years old generate children with low birth weight in 90% of the cases, in addition to premature births^40,41^. In agreement, Katz-Jaffe et al. observed that female CF1 mice superovulated at 6–8 weeks (1–2 months) of age mated with males with more than 12 months of age presented smaller and lighter fetuses; and a decrease in placental weight^37^. Similarly, Denomme et al. verified the same changes in their study, such as the decrease in weight and length of fetuses, lighter placentas and a decrease in successful mating frequency from males’ mice aged 11–15 months^42^.

Paternal age can affect placental development since changes in sperm DNA and epigenetic dysregulation can be frequent in older men^43^. Surprisingly, the diameter, area and weight of the placenta showed no statistical difference, unlike previous studies, which demonstrated that advanced paternal age affects placental development in mice^37,42^ and humans^8,44^. In the human species, the placenta weight from pregnancies with older men (over 50 years old) increased compared to the group between 20 and 24 years old^44^. The consequence of the decrease in human placenta weight would be portrayed as an inadequate exchange of nutrients and gases due to the smaller surface area. On the other hand, an increase in placenta weight could indicate edema of the placental villi which would reduce the transfer of nutrients and gases^45^.

Recent studies indicate that the ratio of human newborn to placental weight may be related to perinatal changes; a higher ratio indicates insufficient oxygen to the fetus and a lower ratio suggests a suboptimal fetal condition^45,46^. In the study by Denomme et al. senile male mice were found to have a higher fetal: placental weight ratio compared to their youth^42^. Controversially, we observed in the present work that senile male mice had a lower fetal: placental weight ratio in relation to the group of young mice, which may be a consequence of the lower fetus weight observed in senile group.

Despite the differences that we observed in the embryo and fetal development, there were no differences on mitochondria function, plasmatic and acrosomal membranes integrity and oxidative stress in young and senile groups. However, we observed lower values for motility, kinetics variables, percentage of rapid sperm; and a higher percentage of static sperm in senile compared to the young group. This result indicated that the sperm of senile males are slower, and probably interferes with sperm fertilization capacity. The positive correlations between the percentages of motile, progress, and rapid sperm with cleavage, blastocyst, and embryo development rates reinforce this hypothesis.

Mitochondrial membrane potential (MMP) correlates positively with sperm parameters such as motility, sperm capacitation, and fertilization^47^. Several studies support that there is a link between aging, mitochondrial dysfunction, and decreased male fertility, as well as changes in fatty acid composition that can alter the fluidity of the inner mitochondrial membrane. In this study we observed a positive correlation between high mitochondrial membrane potential and embryo development rates in the senile group indicating that an increase in the number of sperm with high mitochondrial membrane potential may improve in vitro embryo development rates.

Controversially, Katz-Jaffe et al.^37^ observed no differences in sperm motility in male mice aged 15 months and 8–10 weeks (1–2 months) of age, this result could be explained by the age of the animals used, which were 3 months younger compared to the age of senile male mice used in the present work. According to Dutta and Segunpta et al., every 9125 days of a mouse represents 1 year for men^34^. Therefore, several changes can occur within a relatively short time, such as 3 months. Moreover, Katz-Jaffe et al. used conventional microscopy to assess sperm motility^37^, and we assessed the sperm motility through the CASA system, obtaining greater accuracy and details on sperm movement pattern compared to routine evaluations by light microscopy^48^.

Results of the present study indicate that senile males present a decrease in reproductive performance. In consonance, in humans, paternal age can be associated with increased prevalence of comorbid conditions of urological character (decrease sperm motility, percentage of normal sperm morphology, sperm concentration, and increased sperm DNA fragmentation and ejaculatory dysfunction)^49–51^, which affect reproductive potential, fertility^46^, low conception rates to poor offspring health^52^.

The increase in paternal age compromises the motility, velocity, and coordination of sperm, and negatively influences in vitro embryo development rates and the size of the fetus in mice. Therefore, more studies are necessary to indicate when male murine reproductive senility occurs, and clarify the biological mechanisms involved in the influence of paternal age on embryo and fetal development.

## Methods

**Figure 4:**
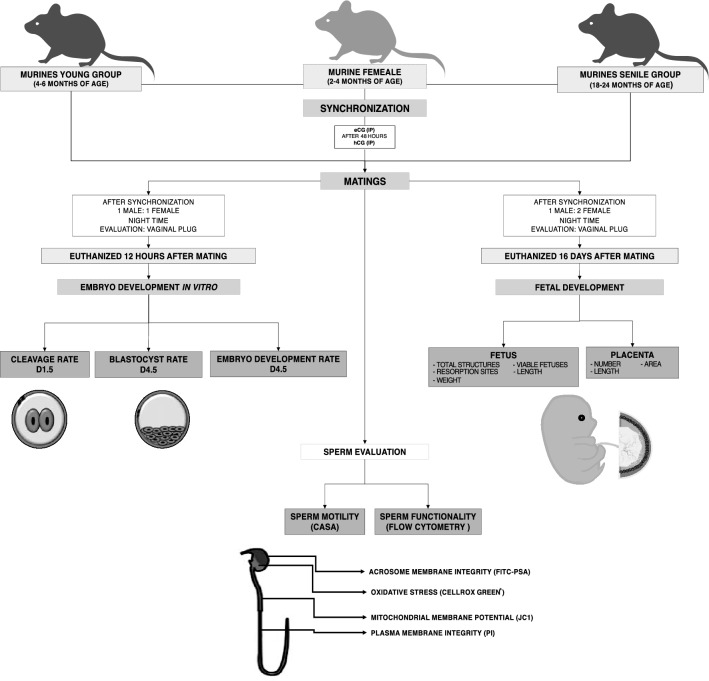
Experimental design. Male murines divides into two groups, senile (18–24 months of age) and young (4–6 months of age), who mated with a group of murine females synchronized, for the realization of three experiments: embryo development in vitro, fetal development, and sperm evaluation.

The present study was conducted following ethical directives for animal experiments, complied with ARRIVE guidelines^53^, and was approved by the Ethics Committee on the Use of Animals of the School of Veterinary Medicine and Animal Science of the University of Sao Paulo (7125160518), and all methods were performed in accordance with the relevant guidelines and regulations. All chemical reagents and solutions used in this study were purchased from Sigma-Aldrich (St. Louis, Missouri, USA) unless otherwise indicated. The experimental design, materials and methods of this work are presented in Fig. 4.

### Animals

We used C57Bl/6J mice from the Laboratory Animal Facility of the Department of Animal Reproduction, School of Veterinary Medicine and Animal Science, University of Sao Paulo. All the animals were maintained in mini-isolators (ALESCO^®^, Sao Paulo, Brazil) at 22–24 °C controlled air temperature, 12 light/12 dark cycle light on room, and offered industrial pellet food and filtered water “ad libitum”.

Male animals were divided into 2 groups, according to their age. The young group was composed of 4–6 months old mice, corresponding to men of approximately 20 years old^34^. The senile group was composed of 18–24 months old animals, corresponding to men of approximately 60–79 years old^34^. As an inclusion factor, we used only senile murine males that did not present neurological, locomotion, ophthalmological, dermatological alterations and visible increases in body volume (tumor or edema), which may be related to metabolic disorders. Before mating, the males remained in groups of up to 4 animals in each mini-isolator, and after mating the males were isolated for the continuation of the other experiments.

We used sexually mature female mice at 2–4 months of age. The females were distributed randomly in the experimental groups and the total number of the experimental units were described in each experiment. During the evaluation of embryo and fetal development, researchers were blinded to the experimental group.

### Murine female management

Females estrous were synchronized with intraperitoneal injection of 5–2.5 IU of eCG (Equine Chorionic Gonadotropin, Novormon, Zoetis, Brazil) followed by 5–2.5 IU of hCG after 48 h, approximately 1 h before the start of the Laboratory Animal Facility dark cycle (12 h).

For the embryo development experiment, we used monogamous mating (1 male:1 female). In the fetal development experiment, monogamous and polygamous (1 male:2 females) mating was performed. For all experiments, after hCG administration, females were transferred to male cage throughout the night, and mating was evaluated in the morning of the following day by visualization of the vaginal plug. Regardless of plug visualization all females were euthanized at the 1^th^ and 16^th^ day of gestation for in vitro embryo and fetal development, respectively.

### Euthanasia procedure

Cervical dislocation was performed in pregnant females after the anesthesia procedure with Isoflurane (BioChimico, Itatiaia, Rio de Janeiro and Cristália^®^, Itapira, Sao Paulo). In the males, cervical dislocation^48^ was performed with no anesthesia to minimize possible seminal alterations. In a parallel experiment (data not showed) we verified sperm aggregation and agglutination when isoflurane was used for the euthanasia procedure.

### Reproductive performance

We calculated the mating rate (number of females with vaginal plug/total number of females that copulated × 100)^55^ per experimental group.

### In vitro embryo development

Experimental groups included 7 males in the senile group and 6 males in the young group, those males were mated monogamously with 13 hormonally synchronized females (2–4 months old). Vaginal plug was not observed in two females of the senile group, with consequent absence of embryos and further embryo development. Therefore, we used 6 hormonally synchronized females for young group and 5 females for senile group, providing 9 degrees of freedom for residue in statistical analyses.

The females were euthanized at 1^th^ day of gestation, the reproductive system was accessed according to Nagy et al.^54^. The oviduct and the attached segment of the uterus were cut and transferred to a 35 mm Petri dish containing HECM-Hepes [(HH)–NaCl 114 mM, KCl 3.2 mM, NaHCO_3_ 2 mM, HEPES 10 mM, Phenol Red, MEM-N.E A.A, Lactic acid, Pyruvic Acid, BSA 0.1 mM, Pen/Step, CaCl_2_.2H_2_0 2 mM, MgCl_2_·6H_2_O 0.5 mM, pH 7.4)])^49^. The presumptive zygotes were released from the oviduct after washing with HH and exposed to a 0.1% hyaluronidase solution in phosphate buffered saline (PBS) to remove cumulus cells, and washed two times in HH plus 5% fetal calf serum (FCS) and then in KSOM medium (MR 020P-5D, Millipore, Massachusetts, USA). Zygotes were IVC in 30 µl drops of KSOM medium, covered with 1–2 ml mineral oil, at 38.5 °C, 5% CO_2_, 5% O_2,_ and 90% N_2_, under high humidity, for 4.5 days.

On day 1.5 of IVC, we assessed cleavage rate (number of cleaved embryos/number of total structures × 100). On day 4.5, blastocyst rate including early blastocyst, expanding blastocyst, expanded blastocyst, hatching blastocyst, and hatched blastocyst (number of blastocysts/number of total structures × 100) and embryo development rate (number of blastocysts/number of cleaved embryos × 100) were assessed. Embryo evaluations were performed in stereomicroscopy (Olympus SZ61, Olympus^®^, Tokyo, Japan) under 60× magnification.

### Fetal development

For fetal development assessment, experimental groups included 20 senile males and 18 young, which were monogamously and polygamously mated with 43 hormonally synchronized females (2–4 months old), 22 females for the senile group and 21 females for young group. Vaginal plug was observed in 10 females mated with senile males and 17 mated with young males. At 16th day of gestation, females mated with senile and young males were euthanized to evaluate the number of total structures, viable fetuses, resorption sites, length and weight of the fetuses, and the area, length and weight of the placenta per male. The litter average from each dependent variable per male was used to perform the statistical analysis. We considered only 5 litters from senile males and 9 litters from young males, therefore 15 senile males and 9 young males did not generate litters. The experiments were conducted with 14 experimental units.

Females were euthanized 16 days after vaginal plug detection. The female reproductive tract was accessed as described previously. Hysterectomy (technique adapted from Olson and Bruce^56^) was performed and the uterus was placed in a 35 mm Petri dish. Uterine horns were sectioned longitudinally, and the content examined. The number of total structures (viable fetus plus resorption sites), viable fetus (compatible with gestational age, E16.5)^57^ and resorption sites were recorded. Fetuses and their extraembryonic tissues were removed, weighed on a digital analytical balance (model AG245, Marshall Scientific®, Hampton, USA), and the weight of fetal and placental ratio.

Photos of fetuses and placentas were taken using a megapixel digital color camera (Olympus LC30, Olympus^®^, Munster, Germany) attached to a stereomicroscope (Olympus SZ61, Olympus^®^, Tokyo, Japan) to measurement of fetal length (crown to rump) and placental length and area were performed by ImageJ Software (Image Processing and Analysis in Java version 1.52 j, public domain, National Institutes of Health, USA) and CellSens^®^ Software (Olympus Live Science^®^, Olympus^®^, Tokyo, Japan).

### Sperm evaluation

Five animals of the senile group and four animals of the young group were randomly selected for the semen evaluation, providing 7 degrees of freedom for residue used in statistical analysis. We performed the power analysis (PROC POWER, SAS System for Windows 9.3) in the sperm variables (CASA and cytometry) to decide the minimal number of the experimental units to provide a power higher than 80%.

Sperm was collected from epididymis cauda and vas deferens according to Yamashiro et al.^58^ and Kishikawa et al.^59^ with modifications. Samples were then added to CZB-Hepes medium [(NaCl 82 mM, KCl 4.9 mM, KH_2_PO_4_ 1.2 mM, MgSO_4_·7H_2_O 1.2 mM, NaHCO_3_ 15 mM, CaCl_2_·2H_2_O 1.7 mM, NaEDTA·2H_2_O 0.004 g, l-Glutamine 1.0 mM, Sodium Lactate Syrup 20 mM, Sodium Pyruvate 0.3 mM, Glucose 5.6 mM, Hepes-Sodium 10 mM, Polyvinyl alcohol, pH 7.4)]^49^. Sperm concentration was assessed using a hemocytometer. Then, we evaluated mitochondrial membrane potential, plasma, acrosome membrane integrity, and oxidative stress by flow cytometry using Guava Easy Cyte™ Mini System (Guava^®^ Technologies, Hayward, CA, USA). We used v8.7 Flowjo software (Flow Cytometry Analysis Software—Tree Star Inc., Ashland, Oregon, USA) to evaluate data.

Mitochondrial membrane potential was accessed by JC-1 probe [(5.5′.6.6′-tetrachloro-1.1′.3.3′-tetraethyl-benzimidazolyl-carbocyanine chloride, 76.5 µM (Invitrogen text superscript^®^, Eugene, OR, USA)] fluorescence, according to Castro et al.^60^. For being a metachromatic probe, mitochondria with low and medium potential fluoresce on green and with high potential fluoresces in red. In a dark room, 0.5 µL of JC-1 (1 µM final concentration) was added to 7.5 µL with 17.5000 sperm and 30 µL of CZB-Hepes medium. Samples were analyzed by flow cytometry after 5 min’ incubation.

Sperm plasma membrane and acrosome integrity was evaluated by flow cytometry using propidium iodide (PI; 0.5 mg/mL, 0.9% NaCl v/v) and fluorescein isothiocyanate conjugated with Pisum sativum agglutinin (FITC-PSA; 100 µg/mL, sodium azide solution at 10%) according to Hamilton et al.^61^. Propidium iodide (PI) fluoresces when it binds to DNA, however, it only penetrates the cell when the membrane is damaged, thus indirectly revealing MP damage, emitting the red fluorescence. While PSA has specificity to acrosome membrane glycoproteins, when conjugated to FITC it marks the damaged acrosome in yellowish-green fluorescence. In a dark room, FITC-PSA solution was prepared to add 190 µL of sodium azide solution at 1% and 10 µL of FITC (final concentration 24.3 µg/mL), then 11.3 µL of PI (final concentration 6.87 µg/mL) was added to solution. About 175,000 sperm were incubated with 13 µL of this solution and 30 µL of CZB-Hepes. Samples were analyzed by flow cytometry after 5 min of incubation. This association of probes separates four sperm populations: intact membrane and intact acrosome (IMIA), intact membrane and damaged acrosome (IMDA), damaged membrane and intact acrosome (DMIA), damaged membrane, and damaged acrosome (DMDA).

The fluorescent probe CellROX green^®^ (Molecular Probes, Eugene, OR, USA) was used to evaluate oxidative stress. CellROX green^®^ quantifies intracellular Reactive Oxygen Species (ROS) when the oxidation occurs, and subsequent binding to DNA, emitting a more intense green fluorescence. According to de Castro et al., in a dark room, 0.6 µL CellROX green^®^ (final concentration 5 µM) was added to 60 µL of CZB-Hepes. About 175,000 sperm were incubated with 1.85 µL of this solution, after 20 min of incubation was added 0.7 µL of PI (final concentration 6.87 µg/mL)^60^. Samples were analyzed by flow cytometry after 10 min of incubation.

Sperm motility was evaluated using computer-assisted sperm analysis (CASA, Hamilton-Thorne Ceros 12.1). Briefly, semen was diluted to a concentration of 25 × 10^6^ sperm/mL, and 10 µL aliquot was inserted (about 1000 sperm cells) were placed on a warmed (37 °C) count chamber (Standart Count 4 chamber slide, 20 microns, Leja^®^, Nieuw Vennep, The Netherlands). For each measurement, at least five different microscopic fields were randomly selected, until reaching 750–1000 sperm cells.

The mean of these scans was used for statistical analysis on Hamilton Thorne IVOS Ultimate 12.3 (Hamilton Thorne Biosciences^®^, Beverly, MA, USA). The variables assessed were: (1) percentages of total motility spermatozoa (TM. %); (2) spermatozoa with progressive motility (PROG. %; (3) average pathway velocity (VAP. µm/s); (4) straight-line velocity (VSL. µm/s); (5) curvilinear velocity (VCL. µm/s); (6) amplitude of lateral head displacement (ALH. µm); (7) beat cross frequency (BCF. Hz); (8) straightness (STR. %) of sperm movement; and (9) linearity (LIN. %). Sperm were also classified based on velocity: rapid (RAP, VAP > 50 µm/s. %); medium (MED, 30 µm/s < VAP < 50 µm/s. %); slow (SLOW, VAP < 30 µm/s or VSL < 15 µm/s. %); and static (STATIC. %), non-moving spermatozoa^62,63^.

### Statistical analysis

Statistical analysis was performed using the Statistical Analysis System 9.3 software (SAS Institute, Cary, NC, USA). The samples were tested to the normality of residues and homogeneity of variances. We performed the T-test procedure for independent variables. A correlation test (Spearman) was performed between sperm traits, embryo development in vivo, and fetal development, considering or not the experimental groups. The probability (p) values will be presented along with the results topic for each variable, considering p significance less than 0.05, to reject the null hypothesis. The data are presented as mean ± SEM (standard error of the mean).

## Supplementary Information

Supplementary Table S1.

Supplementary Table S2.

## Data Availability

The authors confirm that the data supporting the findings of this study are available within the article and its supplementary materials.
